# Construct validity, responsiveness, and interpretability of the Utrecht Scale for Evaluation of Rehabilitation (USER) in patients admitted to inpatient geriatric rehabilitation

**DOI:** 10.1177/02692155231203095

**Published:** 2023-09-25

**Authors:** Margot W M de Waal, Michael Jansen, Loes M Bakker, Arno J Doornebosch, Elizabeth M Wattel, Dennis Visser, Ewout B Smit

**Affiliations:** 1 University Network for the Care sector Zuid-Holland, Leiden University Medical Center, Leiden, the Netherlands; 2 Department of Public Health and Primary Care, Leiden University Medical Center, Leiden, the Netherlands; 3 Faculty of Health, Physiotherapy, University of Applied Sciences Leiden, Leiden, the Netherlands; 4 Woon Zorgcentra Haaglanden (WZH), The Hague, the Netherlands; 5 Department of Medicine for Older People, Amsterdam UMC, Location Vrije Universiteit Amsterdam, Amsterdam, the Netherlands; 6 Amsterdam Public Health Research Institute, Aging & Later Life, Amsterdam, the Netherlands; 7 de Zorgcirkel, Purmerend, the Netherlands; 8 University Network of care for Older people of Amsterdam UMC (UNO Amsterdam), Amsterdam UMC, Amsterdam, the Netherlands; 9 Vivium Zorggroep, Naarden, the Netherlands

**Keywords:** Geriatric rehabilitation, post-acute care, clinimetrics, measurement properties, physical functioning, cognitive functioning

## Abstract

**Objective:**

The Utrecht Scale for Evaluation of Rehabilitation is a multi-domain measurement with good content validity, structural validity and reliability for measuring physical functioning (mobility, selfcare) and cognitive functioning in geriatric rehabilitation. We aimed to determine the construct validity of both Utrecht Scale for Evaluation of Rehabilitation scales and the responsiveness and interpretability of the scale for physical functioning in geriatric rehabilitation.

**Design:**

Prospective follow-up study embedded in routine care.

**Setting:**

Four care organisations in The Netherlands.

**Subjects:**

Patients admitted for inpatient geriatric rehabilitation (2021–2022).

**Main measures:**

Data collection included the Utrecht Scale for Evaluation of Rehabilitation, Mini-Mental State Examination, Barthel index, and a global rating scale anchor on recovery. Hypothesis testing was used to determine construct validity and responsiveness. For interpretability, minimal important change and floor and ceiling effects were determined.

**Results:**

The mean age of participants (*n* = 211) was 77 (SD 10.4). Their mean length of stay was 38.6 days (SD 26.3), and 81% returned home. The Utrecht Scale for Evaluation of Rehabilitation showed adequate construct validity, as all three hypotheses were confirmed for both scales. The Utrecht Scale for Evaluation of Rehabilitation-physical function scale showed adequate responsiveness, with all five hypotheses confirmed. The mean change for physical function (scale range 0–70) was 15.5 points (SD 17.1). The minimal important change for Utrecht Scale for Evaluation of Rehabilitation-physical function was 14.5 points difference for improvement. This scale showed no floor (2%) and ceiling effects (14%) at admission and discharge.

**Conclusions:**

The Utrecht Scale for Evaluation of Rehabilitation showed to be effective for evaluating physical functioning during geriatric rehabilitation as well as screening cognitive functioning. In total, 14.5 points difference has been established as a minimal important change for physical functioning.

## Introduction

A growing number of frail or older people are in need of geriatric rehabilitation, e.g., when faced with a sudden decline in functioning due to acute illness. Patients are characterized by multimorbidity and frailty, and often decreased trainability and learnability.^1,2^ Moreover, cognitive ability influences the effectiveness of rehabilitation.^3^ Measuring physical and cognitive function is essential to optimize treatment.^4,5^

It is important to use measurement instruments with adequate measurement properties for the intended population. This will ensure that an instrument measures what it is meant to measure (validity), can do so reliably (reliability), and it can detect change over time (responsiveness).^6^ Also an adequate distribution of scores (e.g., no ceiling effect) and understanding of the meaning of quantitative scores or change in scores (interpretability) is important. A systematic review showed that various instruments are used for measuring physical functioning in hospitalized older patients, with no conclusive evidence of psychometric superiority for any one instrument.^7^ For physical function, the Katz index and the Barthel index are probably most often used.^8,9^ A recent study in geriatric rehabilitation showed that the Barthel index has good measurement properties except for the disadvantage of its ceiling effect.^8^ Several available instruments to screen for cognitive impairments have good psychometric properties, such as the Mini-Mental State Examination (MMSE) and the Montreal Cognitive Assessment.^10–12^ However, a potential disadvantage is that both need an interview with the patient. There is a need for an observational instrument with adequate psychometric properties.

A promising generic multi-domain instrument is the Utrecht Scale for Evaluation of Rehabilitation (USER), as it contains items for both physical and cognitive function. It is an observational instrument for an objective appraisal of functioning in daily life and as such not burdensome for patients. The USER was developed in the field of adult rehabilitation,^13^ and we recently showed promising measurement properties for the setting of geriatric rehabilitation. In our previous study on the psychometric properties of the USER, the scales on physical and cognitive function showed moderate to excellent content validity, sufficient structural validity, and excellent interrater reliability.^14^ Construct validity, interpretability, and responsiveness have not been established in geriatric rehabilitation.

The objective of this study is to determine the construct validity, responsiveness, and interpretability (distribution of scores and minimal important difference) of the observational scales of the USER in geriatric rehabilitation. For responsiveness and minimal important difference, we only examined the physical function scale, as the cognitive function scale is primarily used for screening purposes at admission.

## Methods

Patients were recruited from inpatient specialized geriatric rehabilitation wards at four care organizations in the Netherlands. The care organizations participate in two academic networks, the University Network of care for Older people (UNO Amsterdam) and the University Network for the Care sector South Holland (UNC-ZH). All participants received regular geriatric rehabilitation, which includes a comprehensive geriatric assessment, the formulation of patient-specific rehabilitation goals, and a multidisciplinary rehabilitation plan fitting the needs of the individual patient.^1^

The study protocol received a waiver of consent from the Medical Ethics Committee of the VU University Medical Centre, Amsterdam, the Netherlands (2020.193). Patients were informed about the study and offered an opt-out to the data being used for this study. Data were collected by health care professionals from April 15, 2021 until March 8, 2022 and passed on to the researchers anonymized and not traceable to any particular person.

Newly admitted patients were all eligible for the study and no selection was applied. The following patient characteristics were retrieved from the electronic patient files: Age, sex, rehabilitation diagnosis, discharge destination, and scores for physical and cognitive function at admittance and discharge. For physical and cognitive function, either USER or Barthel index was part of standard routine care, and the other instrument was added during the study period. In one nursing home, both instruments were standard routine care. In addition, in two nursing homes, a medical student (LB) administered the MMSE during the first week of admittance. In addition to routine care, healthcare professionals registered the Global Rating Scale at discharge.

The USER is an observational instrument that measures physical function (independence in ADL-activities of mobility and selfcare) and cognitive function. The Dutch version 1.4 was used, downloaded from www.kcrutrecht.nl/producten/user/ (see English translation in Supplemental information 1). It consists of 24 observational items: 7 items on mobility, 7 items on self-care, and 10 items on cognitive functioning (communication, cognition, and behaviour). Items are scored on a 6-point scale from 0 to 5. Scores of items on mobility and self-care are added to a total score for USER-physical functioning (USER-PF, range 0–70), and scores of items on cognitive functioning are added to a total score for USER-cognitive function (USER-CF, range 0–50) with lower scores indicating more dependency on others.

The USER was developed as a classic-test theory instrument for adult rehabilitation^13^ and in our previous study we tested its clinometric properties in geriatric rehabilitation.^14^ We used confirmatory factor analysis to test structural validity and unidimensionality, and we confirmed that the observational scales measure two underlying independent constructs, in which the items on mobility and self-care belong to one underlying construct. We further showed sufficient content validity, internal consistency, and interrater reliability in geriatric rehabilitation. For the physical functioning scale, the standard error of measurement (SEM) is 5 and the smallest detectable change (SDC) is 14.^14^ The USER also has a set of patient-reported items (on pain, fatigue, and emotion) that can be left out and were not studied here.

The Barthel index is an observational instrument that measures physical functioning in 10 basic ADL-activities, such as eating, mobility, and grooming.^15^ Items between Barthel index and USER-PF largely overlap (see Supplemental information 2). The Barthel Index total score ranges from 0 to 20. Higher scores indicate a higher level of independence in activities of daily life functioning. The structural validity, reliability, and interpretability of the Barthel index are considered sufficient for measuring and interpreting changes in the physical function of patients in geriatric rehabilitation.^8^ It showed high responsiveness in hospital patients with stroke^16^ and the culturally adapted Italian version (IcaBI) was responsive to the results of inpatient rehabilitation in patients with orthopedic and neurologic diseases.^17^

The MMSE is a widely used screening test to measure cognitive functioning.^18^ The test consists of 11 questions, measuring various aspects of cognitive functioning (attention, memory, language, and visual-spatial skills). The total score ranges from 0 to 30 and higher scores represent a higher level of cognitive functioning, with scores ≥24 classified as ‘no cognitive impairment’. The MMSE has been validated in geriatric patients, and construct validity is satisfactory.^19^

The Global Rating Scale, a generic single-item scale, was used to ask the responsible nurse the following question: ‘To what extent has the physical function of the patient changed during the rehabilitation period?’. There were five response options: (a) much deteriorated; (b) somewhat deteriorated; (c) no change; (d) somewhat improved; (e) much improved.

To be included in the analyses, participants should at least have data on the USER and Barthel index at admission and discharge, or data on USER and Barthel index and MMSE at admission. Because gold standards for measuring or detecting a change in physical and cognitive functioning are lacking in geriatric rehabilitation, construct validity and responsiveness can best be determined by hypotheses testing.^20^ Hypotheses are based on comparing scores between instruments or time points, or by comparing scores between meaningful subgroups.

Construct validity is ‘the degree to which scores of a measurement instrument are consistent with hypotheses, for instance with regard to internal relationships, relationships with scores of other instruments or differences between relevant groups’.^21,22^ Three hypotheses were formulated a priori for each USER subscale (see Table 3). Construct validity was considered to be adequate if two out of three hypotheses were confirmed.^21,23^ Hypotheses were based on the assumption that USER scores would correlate between admission and discharge^24^, and that scores on USER-PF would correlate with Barthel Index^25^ and USER-CF with MMSE. We consider the Barthel Index and USER-PF to measure a related construct, with Pearson's correlation coefficient of *R* > 0.5, as found earlier in adult inpatients with stroke^25^ (deviation from protocol (*R* > 0.3)). Although the MMSE is not an observational instrument we consider the MMSE to measure a similar (related) construct compared to USER-CF, with Pearson's correlation coefficient of *R* > 0.5 (deviation from protocol (*R* > 0.3)). We hypothesize that scores of the same scale at two-time points (admission and discharge) will be highly correlated, with Pearson's correlation coefficient of *R* > 0.5. Differences in USER scores between groups were analyzed using a *t*-test or Mann-Whitney *U* Test where applicable, with *p* ≤ .05 assuming a significant difference.

Responsiveness is ‘the ability of an instrument to detect change over time in the construct to be measured’.^20^ As there is no gold standard available for detecting changes in physical functioning in geriatric patients, hypothesis testing was used. Responsiveness for the USER-PF scale was considered to be adequate if four out of five formulated hypotheses (see Table 2) were confirmed. Hypotheses were based on the assumption that change scores of the USER would correlate with change scores of the BI; and that change scores, effect sizes (ESs), and standardized response means (SRM) would differ between meaningful subgroups of patients. First, it was hypothesized that patients who went home at discharge would have improved more than patients who did not go home at discharge from geriatric rehabilitation. The latter group was formed by patients who went to a care home, nursing home, hospital, hospice, or palliative care at home. Next, anchor groups were formed based on the Global Rating Scale. Patients who were rated as somewhat improved or much improved were classified as ‘improved’, and patients who were rated as somewhat deteriorated or not changed were classified as ‘not improved’. The data of the much-deteriorated patients will not be used for this analysis since we are primarily interested in detecting progress instead of deterioration. ES and SRM were calculated for the two anchor groups: the mean change scores between discharge and admission were divided by the standard deviation (SD) of the baseline score for the ES, or by the SD of the change score for SRM.^26^ Finally, the USER-PF change scores of both anchor groups were compared and used to determine an Area Under the Curve (AUC). Cut-off values for correlations were chosen in the same way as previously described for construct validity.Interpretability is ‘the degree to which one can assign qualitative meaning to instruments’ quantitative scores or change in scores’.^22^ In other words, it is the degree to which it is clear what the scores or change in scores mean. First, the distribution of the scores was examined. Floor and ceiling effects were determined by calculating the percentage of patients with the lowest and highest possible scores at the beginning and end of their rehabilitation period. A significant floor or ceiling effect was considered present if the percentage exceeded 15%.^23^ Next, the minimal important change (MIC) of the USER-PF scale was estimated using predictive modelling. The MIC is defined as ‘the smallest change in score in the construct to be measured which is perceived as important’. The predictive modelling approach is based on the predicted probability that a patient belongs to the improved group given the observed change score and uses logistic regression analysis. First, the sample was divided into two groups based on the Global Rating Scale; (1) patients reported to be somewhat or much improved (improved group) and (2) patients reported to be not changed or somewhat or much deteriorated (not improved group) excluding deceased patients. Next, the MIC was estimated by following instructions on MIC_predict (adjusted)_ in the Appendix from Terwee et al.^27^ The MICs were adjusted for the proportion of improved patients.^28^ The 95% confidence interval around the adjusted MIC was calculated using non-parametric bootstrapping (1000 samples).

R version 3.6.3. (R foundation for statistical computing, Vienna Austria) was used to calculate the MIC confidence intervals. All other analyses were performed using SPSS (IBM SPSS Statistics for Windows, Version 25.0; IBM Corp, Armonk, NY).

## Results

**Figure 1.**
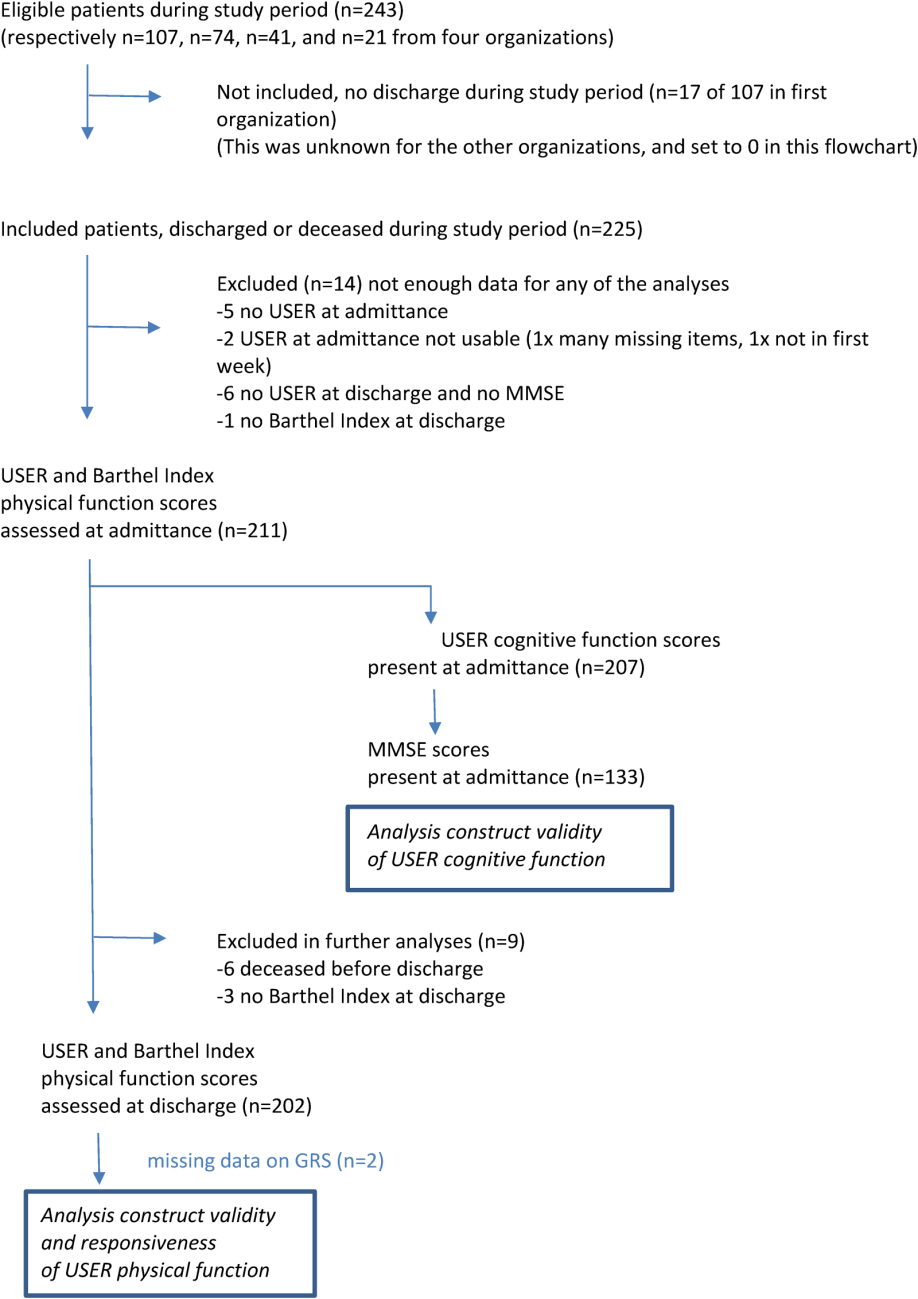
Flowchart of study population.

A total of 225 participants were included in the study, of whom 211 participants could be included in analyses (see Figure 1). For the other 14 participants, too much data was missing. The mean age of the participants was 77 years (SD 10.4). Rehabilitation diagnoses at admission were stroke 19.4%, trauma 2.4%, elective orthopedic surgery 13.7%, amputation 24.2%, or miscellaneous 40.3% (e.g., lung and heart rehabilitation, post-coronavirus disease 2019 (COVID-19) rehabilitation). The mean length of stay was 38.6 days (SD 26.3), and 80.6% returned to their own home. Six patients (2.8%) died during geriatric rehabilitation. At discharge, care professionals reported the majority of participants to be much improved (52.2%) or somewhat improved (28.1%) on the Global Rating Scale (Table 1).

**Table 1.** Patient characteristics of the study population.

	Patients (*n* = 211)	
Age, mean (SD), range	77	(10.4) 40–101
Sex, female, *n* (%)	137	(64.9)
Reason for admittance, *n* (%)		
Stroke	41	(19.4)
Trauma	5	(2.4)
Elective orthopaedic surgery	29	(13.7)
Amputation	51	(24.2)
Miscellaneous	85	(40.3)
Length of stay in days, mean (SD), range	38.6	(26.3) 1–167
Discharge destination		
Home	170	(80.6)
Care home	3	(1.4)
Nursing home (long-term care)	19	(9.0)
Hospital	10	(4.7)
Hospice/palliative care at home	2	(0.9)
Other	1	(0.5)
Deceased	6	(2.8)
Global Rating Scale at discharge^a^, *n* (%)		
Much improved	106	(52.2)
Somewhat improved	57	(28.1)
No change	24	(11.8)
Somewhat deteriorated	6	(3.0)
Much deteriorated	10	(4.9)

a Missing for 2 patients; deceased patients (*n* = 6) excluded.

For a subsample of 133 participants, MMSE was administered at admission, and results were used for the analysis of the construct validity of the USER-CF scale. In this subsample, the median USER-CF scores at admittance were comparable to the whole sample (Table 3). For a subsample of 202 participants, follow-up data on USER and Barthel index were available, which allowed for analyses of construct validity and responsiveness of the USER-PF scale. In this subsample, the USER scores at admittance were comparable to the whole sample (Table 3).

Table 2 presents the results of the hypothesis testing. The USER-PF scale showed adequate construct validity. All three hypotheses were confirmed. The USER-CF scale also showed adequate construct validity. Again, all three hypotheses were confirmed. The USER-PF scale showed adequate responsiveness. All five hypotheses were confirmed. Participants with follow-up (*n* = 202) improved on average by 15.5 points (SD 17.1) on USER-PF (scale range 0–70) (Table 3). Improvement was significantly higher (*p* < 0.001) in the group that showed important change compared to the group that did not show important change, with mean change scores on USER-PF of 19.0 (SD = 15.3; *n* = 163) and 7.4 (SD = 14.5; *n* = 30), respectively. For the groups that did or did not return home mean change scores were 18.5 (SD 15.2; *n* = 168) and 0.65 (SD = 18.9; *n* = 34), respectively (*p* < 0.001).

**Table 2.** Hypotheses testing for construct validity and responsiveness.

		Result	Confirmed hypothesis
	**Construct validity - USER cognitive function scale**	(*n* = 133)	
1	Correlation between scores on USER cognitive function scale and MMSE at admission: Pearson *R* > 0.50	*R* = 0.48	Yes
2	Patients with MMSE ≤ 24 have significant lower scores on USER cognitive function scale than patient with MMSE > 24 (Mann Whitney U Test, *p* ≤ 0.05)	*P* < 0.001	Yes
3	Correlation between scores on USER cognitive function scale at admission and discharge: Pearson *R* > 0.5	*R* = 0.54	Yes
	**Construct validity – USER physical function scale**	(*n* = 202)	
4	Correlation between scores on USER physical function scale and Barthel index, at admission: Pearson R > 0.5	*R* = 0.76	Yes
5	Correlation between scores on USER physical function scale and Barthel index, at discharge Pearson *R* > 0.5	*R* = 0.91	Yes
6	Correlation between scores on USER physical function scale at admission and at discharge; Pearson *R* > 0.5	*R* = 0.55	Yes
	**Responsiveness – USER physical function scale**		
7	The change score on the USER physical function scale correlates with the change on the Barthel index, with Pearson *R* > 0.50	(*n* = 202) *R* = 0.65	Yes
8	The mean change score on the USER of patients returning home is significantly higher than the mean change score of patients who are not able to return to home^a^ (*p* ≤ 0.05, *t*-test)	(*n* = 202) *P* < 0.001	Yes
9	ES and SRM ≥ 1.0 for the improved patient group^b^	(*n* = 163) ES = 1.16 SRM = 1.24	Yes
10	ES and SRM < 1.0 for the not improved patient group^b^	(*n* = 30) ES = 0.31 SRM = 0.51	Yes
11	AUC > 0.7	(*n* = 193) AUC = 0.73	Yes

AUC: area under the curve; ES: effect size; SRM: standardized response mean; USER: Utrecht Scale for Evaluation of Rehabilitation.

a Patients who died during their stay were excluded from this analysis.

b Improved when Global Rating Scale ‘somewhat improved (++)’ or ‘much improved (+)’, not improved when Global Rating Scale ‘somewhat deteriorated (-)’ or ‘not changed (0)’. Patients with Global Rating Scale ‘much deteriorated (- -)’ were excluded.

**Table 3.** Scores at baseline (admittance) and follow-up (discharge) on physical and cognitive functioning during stay in geriatric rehabilitation.

	Admittance (*n* = 211)	Admittance; subsample with follow-up^a^ (*n* = 202)	Discharge (*n* = 202)	Change scores (*n* = 202)
USER physical function (0–70)				
Mean (SD)	37.1 (18.0)	37.9 (17.7)	53.4 (18.6)	15.5 (17.1)
Median (P25–P75)	38 (24–52)	39 (24.8–52.3)	61 (48–67)	13 (3–28)
Range	0; 70	0; 70	0; 70	−51; 62
Minimum score, *n* (%)	1.4%	1.5%	1.0%	
Maximum score, *n* (%)	1.9%	2.0%	13.9%	
Barthel index (0–20)				
Mean (SD)	11.0 (5.0)	11.2 (5.0)	15.5 (5.2)	4.3 (4.3)
Median (P25–P75)	11 (8–15)	11 (8–15)	17 (13.8–19)	4 (1–7)
Range	0; 20	0; 20	0; 20	−9; 19
Minimum score, *n* (%)	2.4%	2.5%	1.5%	
Maximum score, *n* (%)	3.8%	4.0%	20.3%	
	Admittance (*n* = 207)	Admittance; subsample with MMSE^a^ (*n* = 133)		
USER cognitive function (0–50)				
Median (P25–P75)	47 (38–50)	49 (42–50)		
Range	0; 50	0; 50		
Minimum score, *n* (%)	1.4%	2.3%		
Maximum score, *n* (%)	37.0%	42.1%		
MMSE score (0–30)	N/A			
Median (P25–P75)		26 (22–28)		
Range		6; 30		
Minimum score, *n* (%)		0%		
Maximum score, *n* (%)		6.8%		

MMSE: Mini-Mental State Examination; N/A: not applicable; USER: Utrecht Scale for Evaluation of Rehabilitation.

a Excluding deceased patients.

Table 3 presents baseline and follow-up scores (that is scores at admittance and discharge) on the USER, together with scores on Barthel index and MMSE. Scores on USER-PF and Barthel Index were normally distributed at admittance but skewed towards better functioning at discharge. At discharge, 14% of participants had the maximum score on USER-PF; for Barthel index this was 20%. The MIC_predict (adjusted)_ of the USER-PF scale was 14.5 (95%CI; 13.5–15.4) (*n* = 202). Scores at admission on USER-CF and MMSE were skewed towards better functioning, as 42% and 7% (of *n* = 133) respectively reached the maximum score.

## Discussion

In this study, important clinical measurement properties of the USER were studied in geriatric rehabilitation. The construct validity was found to be adequate for the observational subscales for physical and cognitive functioning, as was the responsiveness of USER-PF. The MIC for USER-PF was found to be 14.5 points, and there were no significant floor or ceiling effects at either admission or discharge.

So far, the responsiveness of the USER has only been studied in hospital populations receiving adult rehabilitation. High ESs were found in rehabilitation inpatients with a mean age of 54 years (SD 14.9)^13^ and in patients with stroke with a mean age of 62 years (SD 11.8).^25^ The latter study found comparable ESs for USER-PF and BI, 0.86 and 0.94 respectively. However, an effect size only has meaning as a measure of responsiveness if we know (or assume) beforehand what the magnitude of the effect of the intervention is.^20^ With hypotheses testing, we showed that USER-PF was valid and responsive to change in a geriatric population, with 14.5 points difference as MIC. In our previous study, we found the SEM to be 5 and the SDC to be 14.^14^ This means that a change of 14.5 points is beyond the measurement error and represents a MIC. We found no ceiling or floor effects either at admission or at discharge. At discharge, 14% of participants had maximum scores on USER-PF. The Barthel index did show a significant ceiling effect at discharge in our study (20%) which is in line with previous research among geriatric rehabilitation inpatients, where Barthel index also showed a ceiling effect of 22% at discharge.^8^

As lower cognitive functioning has consequences for available treatment options and is considered to be related to unsuccessful rehabilitation,^5^ it is thus important to screen for cognitive functioning in an early stage of geriatric rehabilitation. We showed that the USER-CF observation scale can be used for this purpose, as construct validity was adequate. This is in line with studies on adult rehabilitation, as the correlation of USER-CF with the functional independence measure (FIM) cognition score was high (0.8) in the development study of the USER.^13^ The USER-CF scale displayed a significant ceiling effect, but as it is used for screening purposes, we do not consider this to be a problem because we are mainly interested in cognitive disfunction. Each score below the maximum of 50 could be a trigger for additional diagnostics. In case of cognitive disfunction, successful rehabilitation is still possible, but this has to be done by changing the rehabilitation style, e.g., by offering an appropriate programme based on errorless learning.^29^ Also, the use of observation instead of interviews is a less burdensome option for both patient and healthcare professional in the research setting.

A possible limitation of our study is, that the study population comprised a relatively low percentage of patients with trauma and a relatively high percentage with amputations, compared to Dutch data.^30^ This was unintentional, and a consequence of participating wards. We did not collect data on patients who were not yet discharged at the end of the study period, inhibiting us to perform a loss-to-follow-up analysis. On the other hand, the length of stay and the proportion of participants that returned to their own home were as expected, so we do not consider this a problem that influenced the outcomes. For 14 (6%) out of 225 included patients we had missing data, which we consider to be missing at random.

A strength of our study is, that we followed routine care as much as possible during data collection, and USER scores were registered by healthcare professionals reflecting usual care. And although it is possible that not all comparison measurements were assessed at the exact same time point as the USER, we do not think this has influenced the results. Another strength is that we assessed measurement properties by testing predefined hypotheses, which are in accordance with COSMIN criteria in the absence of a gold standard. However, we acknowledge the challenges of hypothesis testing which is widely debated in the clinometric literature and community.^20^ In fact, we changed two pre-defined hypotheses (before performing analyses) after careful discussions.

In conclusion, in a consecutive set of studies described in this paper and in a previous one,^14^ we established that the USER has good measurement properties for screening cognitive functioning as well as evaluating physical functioning during geriatric rehabilitation. We therefore recommend the use of the USER in geriatric rehabilitation.

**Clinical messages**

- The USER observational subscales show adequate construct validity for physical and cognitive functioning in geriatric rehabilitation.
- The USER physical functioning scale is responsive to change, with 14.5 points difference as a MIC.
- We recommend the use of the USER for geriatric rehabilitation inpatients.

## Supplemental Material

sj-pdf-1-cre-10.1177_02692155231203095 - Supplemental material for Construct validity, responsiveness, and interpretability of the Utrecht Scale for Evaluation of Rehabilitation (USER) in patients admitted to inpatient geriatric rehabilitation Click here for additional data file. Supplemental material, sj-pdf-1-cre-10.1177_02692155231203095 for Construct validity, responsiveness, and interpretability of the Utrecht Scale for Evaluation of Rehabilitation (USER) in patients admitted to inpatient geriatric rehabilitation by Margot W M de Waal, Michael Jansen, Loes M Bakker, Arno J Doornebosch, Elizabeth M Wattel, Dennis Visser and Ewout B Smit in Clinical Rehabilitation
