# Supplementary material for: Construct validity, responsiveness, and interpretability of the Utrecht Scale for Evaluation of Rehabilitation (USER) in patients admitted to inpatient geriatric rehabilitation
Source: Clin Rehabil. 2023 Sep 25;38(1):98–108. doi: 10.1177/02692155231203095 (PMC10631283; doi:10.1177/02692155231203095)
Supplement: sj-pdf-1-cre-10.1177_02692155231203095 - Supplemental material for Construct validity, responsiveness, and interpretability of the Utrecht Scale for Evaluation of Rehabilitation (USER) in patients admitted to inpatient geriatric rehabilitation [file sj-pdf-1-cre-10.1177_02692155231203095.pdf]

Utrecht Scale for Evaluation of Rehabilitation (USER)

Patientnumber ______________ Patient ____________________________ Assessor ____________________ Date __________

Type of measurement: measurement / goal * intake/ intermediate /discharge * ** circle what applies*

Mobility

	Score (0-5)
1. Sitting	______
2. Standing	______
3. Transfers	______
4. Walking indoors	______
5. Walking longer distances	______
6. Climbing stairs	______
7. Wheelchair mobility *(Note: divergent scoring)*	______

Score Mobility ______ / 35

Selfcare

8. Eating and drinking	______
9. Grooming	______
10. Showering/ Bathing	______
11. Dressing/ Undressing	______
12. Toileting/ bladder voiding and defecation	______
13. Bladder incontinence *(Note: divergent scoring)*	______
14. Fecal incontinence *(Note: divergent scoring)*	______

Score Selfcare ______ / 35

Communication

	Score (0-5)
15. Expressing oneself	______
16. Comprehension	______

Cognition

17. Visual perception	______
18. Orientation in space and time	______
19. Attention and concentration	______
20. Memory	______
21. Task execution	______

Behaviour

22. Initiative	______
23. Behaviour control	______
24. Social behaviour	______

Score Cognitive functioning ______ / 50

Ask the patient to indicate how he/she felt the last few days, regarding:

25. Pain

None at all	0	10	20	30	40	50	60	70	80	90	100	Worst imaginable

26. Fatigue

None at all	0	10	20	30	40	50	60	70	80	90	100	Worst imaginable

27. Depressed mood

None at all	0	10	20	30	40	50	60	70	80	90	100	Worst imaginable

28. Grief

None at all	0	10	20	30	40	50	60	70	80	90	100	Worst imaginable

29. Anxiety

None at all	0	10	20	30	40	50	60	70	80	90	100	Worst imaginable

30. Anger

None at all	0	10	20	30	40	50	60	70	80	90	100	Worst imaginable

Score Mood = depressed mood + grief + anxiety + anger = / 400

USER version 1.4, May 2015

© De Hoogstraat, Kenniscentrum Revalidatiegeneeskunde Utrecht

Scoring system of the USER 1.4

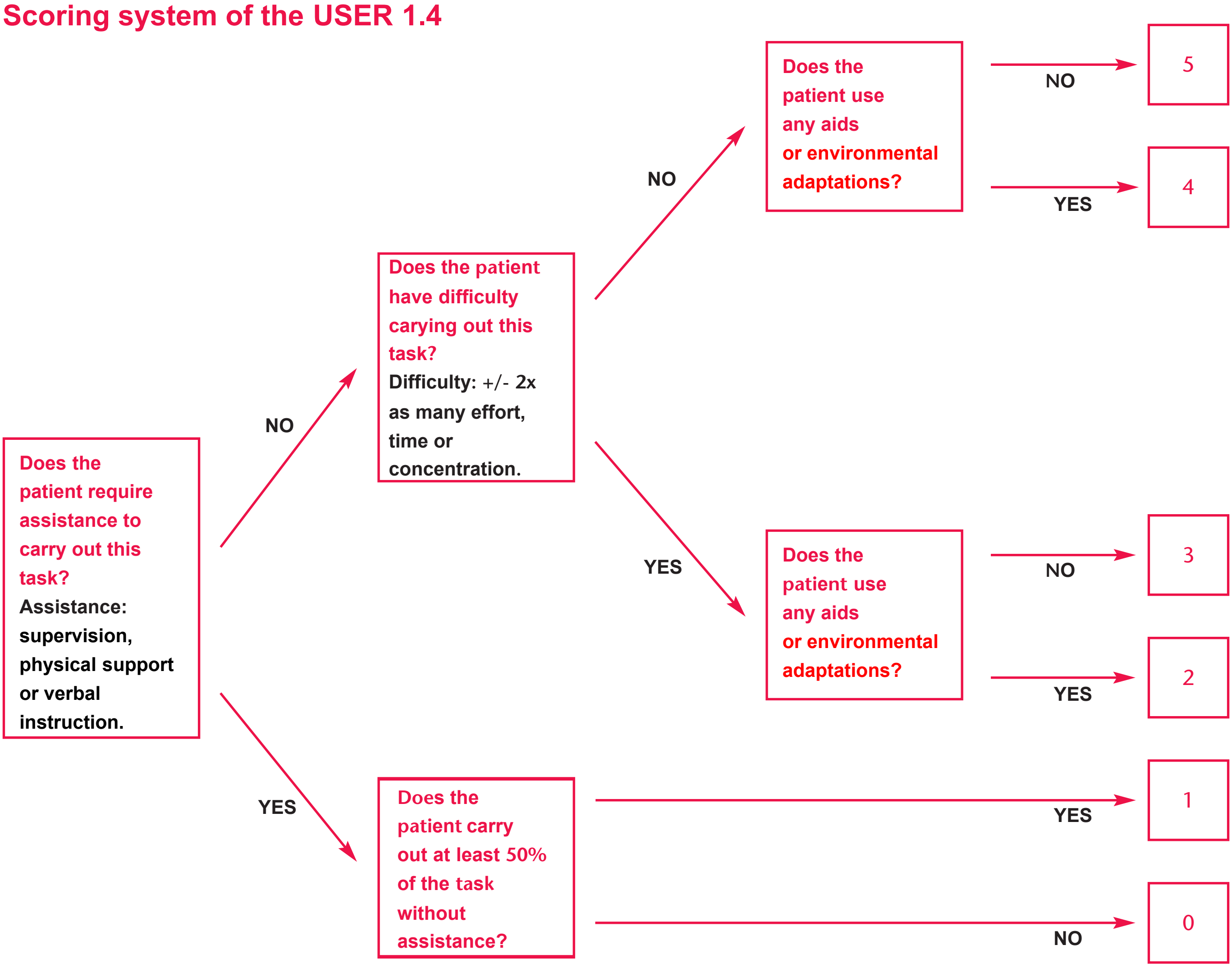

Tips for scoring

- Make use of the manual (www.dehoogstraat.nl/meetinstrument-user)
- Score what the patient actually does in every day life at the rehabilitation department
- When in doubt consult a colleague
- If a patient's level of functioning varies with the situation or over time, you should record the lowest score when in doubt

Scoring for assistance, difficulty or aids

5 = without difficulty, without assistance, aids or adaptations
4 = without difficulty, without assistance, with aids or adaptations

3 = with difficulty, without assistance, without aids or adaptations
2 = with difficulty, without assistance, with aids or adaptations

1 = supervision and/or partial assistance of others (>50% self)

0 = largely or completely by others (< 50% self), or not executed

Divergent scores

Wheelchair mobility
5 = hand-propelled wheelchair without difficulty, or no wheelchair
3 = hand-propelled wheelchair, with difficulty
1 = electric wheelchair
0 = driving wheelchair not possible without assistance
Note: difficulty also means: only short distances, with some assistance in difficult circumstances *(e.g. doorsill)*

Frequency of incontinentie
5 = continent for urine or faeces
4 = continent for urine or faeces with aids
(stoma, catheter)
3 = less than 1x per week incontinent
1 = 1-7 x per week
0 = more than 1x per day

Construct validity, responsiveness, and interpretability of the Utrecht Scale for Evaluation of Rehabilitation (USER) in patients admitted to inpatient geriatric rehabilitation.

Supplement table. Comparison of items on physical functioning of USER with Barthel Index

	**USER items**	**Barthel Index items (order of items)**
mobility	Sitting	
	Standing	
	Transfer	Transfers (6)
	Walking indoors	Mobility (7)
	Walking longer distances	
	Climbing stairs	Stairs (9)
	Wheelchair mobility	Mobility (7)
selfcare	Eating and drinking	Feeding (5)
	Grooming	Grooming (3)
	Bathing/showering	Bathing (10)
	Dressing/undressing	Dressing (8)
	Toileting/ bladder voiding and defecation	Toilet use (4)
	Bladder incontinence	Incontinence urine (controlling bladder) (2)
	Faecal incontinence	Incontinence feces (controlling bowel) (1)
